# Two-stage procedures for selecting the best diagnostic biomarkers

**DOI:** 10.1098/rsta.2008.0032

**Published:** 2008-04-11

**Authors:** Aiyi Liu, Chengqing Wu, Kai F. Yu

**Affiliations:** 1 Biometry and Mathematical Statistics Branch, National Institute of Child Health and Human Development6100 Executive Boulevard, Rockville, MD 20852, USA; 2 Department of Epidemiology and Public Health, Yale University School of MedicineNew Haven, CT 06520, USA

**Keywords:** classification rate, diagnosis, nuisance parameters, probability of correct selection, sensitivity, specificity

## Abstract

Considered in the paper is the problem of selecting a diagnostic biomarker that has the highest classification rate among several candidate markers with dichotomous outcomes. The probability of correct selection depends on a number of nuisance parameters from the joint distribution of the biomarkers and thus can be substantially affected if these nuisance parameters are misspecified. A two-stage procedure is proposed to compute the needed sample size that achieves the desired level of correct selection, as so confirmed by simulation results.

## 1. Introduction

In the area of diagnostic medicine, a biomarker is used to identify subjects that have a certain condition, e.g. a disease or risk status for the disease, so that proper treatments can be followed (Zhou *et al*. 2002). Often, multiple candidate biomarkers are developed simultaneously and medical investigators are interested in selecting the one with the highest diagnostic accuracy among these newly identified biomarkers so that future studies can be focused on the selected biomarker.

One approach to achieving this goal is as follows. A number of subjects are randomly selected from a population with the condition and one without the condition, hereafter referred to as ‘diseased’ and ‘non-diseased’ populations, respectively. The candidate biomarkers are then tested on each subject and their diagnostic accuracy is evaluated from the test results. The biomarker that yields the highest (estimated) accuracy will then be identified as the best biomarker.

A key feature associated with such a selection process is the probability of correct selection (PCS), that is, the probability that the selected biomarker indeed has the highest diagnostic accuracy. The number of diseased and non-diseased subjects needs to be sufficiently large so that the PCS can be maintained at a certain desired level. Since the biomarkers are tested on the same subject, their test outcomes from the same subject are correlated, following a multivariate distribution. Thus, the PCS depends on a number of parameters from the joint distribution which have to be specified in order to have an estimate of the sample size. Unfortunately, however, the PCS can be substantially adversely affected if these parameters are not correctly quantified.

To relax the dependence of the PCS on these parameters, we propose a two-stage procedure. First, we randomly select a number of diseased and non-diseased subjects and use the test results from these subjects to estimate the corresponding parameters in the PCS function. Then, the total number of diseased and non-diseased subjects is computed using these estimates that achieve the desired PCS level when the diagnostic accuracy of the best biomarker differs from the other biomarkers by a specified amount. Simulation results are presented to demonstrate the effectiveness of the proposed procedure in achieving the PCS. This paper ends with some discussions in §6.

## 2. Formulation of the problem

Suppose there are *K* candidate biomarkers under consideration, and each yields a binary test result on a subject, 1 or 0, representing the subject being classified as diseased or non-diseased, respectively, by the biomarker. To assess their diagnostic accuracy, these biomarkers are tested on a random sample of *m* diseased and *n* non-diseased subjects. For the *k*th biomarker, its test result is denoted by *X*_*ik*_ from the *i*th diseased subject and *Y*_*jk*_ from the *j*th non-diseased subject. With these notations, the sensitivity (the probability of correctly classifying a diseased subject) of the *k*th biomarker is $p_{k}=P\{X_{ik}=1\}$ and can be estimated by ${\hat{p}}_{k}=\sum_{i=1}^{m}X_{ik}/m$. Its specificity (the probability of correctly classifying a non-diseased subject) is $P\{Y_{jk}=0\}=1-q_{k}$, with $q_{k}=P\{Y_{jk}=1\}$ and can be estimated by $1-{\hat{q}}_{k}$, with ${\hat{q}}_{k}=\sum_{j=1}^{n}Y_{jk}/n$. The classification rate *θ*_*k*_ of the *k*th biomarker is the sum of the two quantities, i.e. $\theta_{k}=p_{k}-q_{k}+1=P\{X_{ik}=1\}-P\{Y_{jk}=1\}+1$, and can be estimated subsequently by ${\hat{\theta }}_{k}=\left(\sum_{i=1}^{m}X_{ik}\right)/m-\left(\sum_{j=1}^{n}Y_{jk}\right)/n+1$. The best biomarker is the one with the largest *θ*, and the one that yields the largest *θ*-estimate will be chosen as the ‘best’ biomarker. Selection error occurs when the largest *θ*-estimate is not from the biomarker with the largest *θ*.

Without loss of generality, we assume that the first biomarker is the best among the candidate markers, i.e. $\theta_{1}={\text{max}}_{k\geq 1}\theta_{k}$. The *l*th biomarker is selected as the best if ${\hat{\theta }}_{l}={\text{max}}_{k\geq 1}{\hat{\theta }}_{k}.$ Therefore, the PCS is

$$\rho =P\left\{{\hat{\theta }}_{1}=\underset{k\geq 1}{\text{max}}{\hat{\theta }}_{k}|\theta_{1}=\underset{k\geq 1}{\text{max}}\theta_{k}\right\}.$$

The key issue is to determine *m* and *n* so that the PCS will be at least *γ*, a specified level of probability, under certain parametric configuration of the *θ*s. Usually we require that *ρ*≥*γ* when $\theta_{1}-{\text{max}}_{k\geq 2}\theta_{k}\geq \Delta$, where *Δ*>0 is also a prespecified constant. For sufficiently large *m* and *n*, ${\hat{\theta }}_{k}(k=1,\ldots ,K)$ follows asymptotically a multivariate normal distribution. Thus if *ρ*=*γ* for $\theta_{1}-{\text{max}}_{k\geq 2}\theta_{k}=\Delta$, then *ρ*≥*γ* for $\theta_{1}-{\text{max}}_{k\geq 2}\theta_{k}\geq \Delta$. Therefore, the sample sizes *m* and *n* are computed by solving the equation

$$P\left\{{\hat{\theta }}_{1}=\underset{k\geq 1}{\text{max}}{\hat{\theta }}_{k}|\theta_{1}-\underset{k\geq 2}{\text{max}}\theta_{k}=\Delta \right\}=\gamma .$$

In order to compute the sample sizes, parameters other than *Δ* which appear in the equation are given values based on ‘educational guess’ or other data sources if possible.

## 3. Computation of PCS

The test outcomes of the *K* biomarkers from a diseased or non-diseased subject follow a multinomial distribution. Exact calculation of the PCS involves the joint probabilities of {*X*_*i*1_=0 or 1, …, *X*_*iK*_=0 or 1} and {*Y*_*j*1_=0 or 1, …, *Y*_*jK*_=0 or 1}, which becomes extremely complicated when *K* is relatively large. Instead, we may use normal approximation to relax the computational complexity, assuming that the sample sizes are relatively large.

Write $\theta =(\theta_{1},\ldots ,\theta_{K})$ and $\hat{\theta }=({\hat{\theta }}_{1},\ldots ,{\hat{\theta }}_{K})$. Then by classical asymptotic theory (Bickel & Doksum 1977), $\hat{\theta }$ follows asymptotically a multivariate normal distribution with mean vector *θ* and a variance-covariance matrix given by

$$\text{var}({\hat{\theta }}_{k})=\frac{1}{m}p_{k}(1-p_{k})+\frac{1}{n}q_{k}(1-q_{k})$$

and

$$\text{cov}({\hat{\theta }}_{k},{\hat{\theta }}_{l})=\frac{1}{m}(p_{k,l}-p_{k}p_{l})+\frac{1}{n}(q_{k,l}-q_{k}q_{l}),$$

where $p_{k,l}=P\{X_{1k}=X_{1l}=1\},q_{k,l}=P\{Y_{1k}=Y_{1l}=1\}$.

Define $\delta_{k}=\theta_{k+1}-\theta_{1}$ and ${\hat{\delta }}_{k}={\hat{\theta }}_{k+1}-{\hat{\theta }}_{1}$, for k=1,…,K−1, and write $\delta =(\delta_{1},\ldots ,\delta_{K-1})$ and $\hat{\delta }=({\hat{\delta }}_{1},\ldots ,{\hat{\delta }}_{K-1})$. Then $\hat{\delta }$ is also normally distributed with mean vector *δ* and variance-covariance matrix $\Sigma =(\sigma_{kl})$, where

$$\sigma_{kk}=\text{var}({\hat{\delta }}_{k})=\frac{1}{m}\left\{p_{k+1}+p_{1}-2p_{1,k+1}-{\left(p_{1}-p_{k+1}\right)}^{2}\right\}+\frac{1}{n}\left\{q_{k+1}+q_{1}-2q_{1,k+1}-{\left(q_{1}-q_{k+1}\right)}^{2}\right\}$$

and

$$\sigma_{kl}=\frac{1}{m}\left\{p_{k+1,l+1}-p_{1,k+1}-p_{1,l+1}+p_{1}-(p_{1}-p_{k+1})(p_{1}-p_{l+1})\right\}+\frac{1}{n}\left\{q_{k+1,l+1}-q_{1,k+1}-q_{1,l+1}+q_{1}-(q_{1}-q_{k+1})(q_{1}-q_{l+1})\right\}.$$

Let $F(x_{1},\ldots ,x_{K-1};\delta ,\Sigma )$ be the multivariate normal distribution function with mean *δ* and variance-covariance matrix *Σ*, then the PCS is approximately

(3.1) $$\rho (\delta ,\Sigma )=P\left\{{\hat{\delta }}_{k}\leq 0,k=1,\ldots ,K-1\right\}=F(0,\ldots ,0;\delta ,\Sigma ).$$

The sample sizes *m* and *n* are computed from the equation F(0,…,0;δ,Σ)=γ under the constraints that *δ*_*k*_=*Δ* for all k=1,…,K−1.

## 4. A two-stage procedure

The asymptotic formula (3.1) of the PCS involves *K*(*K*+1) parameters $\left\{p_{k},q_{k},p_{k,l},q_{k,l}:1\leq k,l\leq K,l\neq k\right\}$ from the multinomial distributions. With the *K*−1 constraints *δ*_*k*_=*Δ*, there are *K*^2^+1 distributional parameters (referred to as nuisance parameters) left to be specified in order to solve for sample sizes, assuming that *m*/*n*=*λ* is a known constant. If these nuisance parameters are misspecified, then the PCS can be substantially adversely affected, resulting in an unacceptably high level of selection error; see §5 for numerical demonstration. In order to maintain the desired level of the PCS, we propose, following Stein's (1945) idea, a two-stage selection procedure to compute the required sample size *N*=*m*+*n*=(1+*λ*)*n* with ratio *λ*=*m*/*n* fixed.

*Step 1*. Randomly select *m*_1_ diseased and *n*_1_ non-diseased subjects from the target populations and test the biomarkers on these subjects. Use the test outcomes *X*_*ik*_, *i*=1, …, *m*_1_ and *Y*_*jk*_, *j*=1, …, *n*_1_ to obtain empirical estimates of the nuisance parameters, given as follows:

(4.1) $${\hat{p}}_{k}=\frac{1}{m_{1}}\sum_{i=1}^{m_{1}}X_{ik},\;\;{\hat{q}}_{k}=\frac{1}{n_{1}}\sum_{j=1}^{n_{1}}Y_{jk},$$

(4.2) $${\hat{p}}_{k,l}=\frac{1}{m_{1}}\sum_{i=1}^{m_{1}}X_{ik}X_{il},\;\;{\hat{q}}_{k,l}=\frac{1}{n_{1}}\sum_{j=1}^{n_{1}}Y_{jk}Y_{jl}.$$

*Step 2*. Plug these estimates into the PCS formula and then solve for sample size *N*′ from the equation *ρ*=*γ*. The required sample size *N* is then set to max(*N*_1_, *N*′), with *N*_1_=*m*_1_+*n*_1_.

The sample size *N* computed in this way is no longer a fixed quantity. Rather, it is a random variable, a statistic of the data from the first stage. Note that the estimates in (4.1) and (4.2) are consistent estimators of the corresponding parameters. For sufficient sample size *N*_1_, the PCS is expected to be approximately *γ* regardless of the true values of the nuisance parameters; see simulation results below.

## 5. Numerical evaluation

Numerical studies are conducted to investigate the effects of the nuisance parameters on PCS and the performance of the proposed two-stage procedure in maintaining the level of PCS. To reduce the computational burden, we use three biomarkers (*K*=3) for illustration.

Table 1 presents the values of PCS under various configurations of the parameter space which determine the joint distribution of the three biomarkers, that is, the joint probability of $p_{ijk}=P\{X_{11}=i,X_{12}=j,X_{13}=k\}$ and $q_{ijk}=P\{Y_{11}=i,Y_{12}=j,Y_{13}=k\}$, for *i*, *j*, *k*=0, 1. These joint probabilities thus give the marginal probabilities, for example,

$$\begin{array}{c} p_{1,2}=P\{X_{11}=1,X_{12}=1\}=p_{111}+p_{110}\\ p_{1}=P\{X_{11}=1\}=p_{111}+p_{110}+p_{101}+p_{100}, \end{array}$$

and so on.

All configurations in table 1 correspond to a fixed value of 0.1 for $\Delta (=\theta_{1}-\theta_{2}=\theta_{1}-\theta_{3})$. The first row in the table specifies the parameters used to compute the sample size, for a single-stage procedure, assuming that *λ*=*m/n*=1. It turns out that, with this configuration, 102 diseased and 102 non-diseased subjects are needed to achieve a PCS of at least 0.95.

The *ρ*-values, computed using formula (3.1), in the remaining rows of table 1 are the PCS with 102 diseased and 102 non-diseased subjects under other specifications of the parameter values. We see that the PCS can be substantially lower than the desired level of 0.95 if the parameters differ from the specified values in the first row of the table. For example, if the corresponding parameters from the joint distribution of the three biomarkers take values from the second row rather than as being specified in the first row of the table, then the PCS can be decreased by more than 20%!

To investigate whether the proposed two-stage procedure can relax the dependence on nuisance parameters and maintain the PCS at the desired level, we conducted 10 000 simulations for each configuration of the parameter space in table 1. The simulated values of PCS are presented as ρ^*^ in the last column of the table. For the first-stage sample size, we used *m*_1_=75 diseased and *n*_1_=75 non-diseased subjects to estimate the corresponding nuisance parameters.

The results in table 1 clearly show that the PCS is maintained at the desired level of 0.95, regardless of the parametric configuration under which the data are generated. Thus the two-stage procedure is proved to be efficient in controlling selection error rates. Numerical results (not shown here) for other configurations and ratios of *λ* reveal similar findings. With such a multidimensional parameter space, however, it is difficult to characterize any configurations for which the two-stage procedure would perform relatively better.

## 6. Discussion

In this paper, we proposed a two-stage procedure to select the best diagnostic biomarker among a number of candidate markers, and numerically demonstrated its effectiveness in controlling the selection error. While there is no universal standard on how the first-stage sample size is determined, it should be relatively large so that accurate estimates of the nuisance parameters can be obtained. On the other hand, it should not be too large when taking the cost effectiveness of a study into consideration.

We confine our attention to biomarkers with dichotomous outcomes. However, the two-stage approach proposed in this paper can be easily extended to biomarkers with ordinal or continuous test results. In these two cases, an appealing selection criterion is to select the marker that has the largest area under its receiver operating characteristic curve (Hanley 1989).

Various selection problems and related procedures have been discussed extensively in the statistical literature (see Bechhofer *et al*. 1968; Gibbons 1988), but mostly focusing on multivariate normal distributions. Applications of the selection concepts and theory to the area of diagnostic medicine are relatively new, and many other issues remain to be solved such as selecting a fixed number of best diagnostic biomarkers and selecting the best biomarker (or all biomarkers) that are better than a standard biomarker. Further research efforts are much needed along these lines.

Throughout this paper we assume that the data will be available for all biomarkers from each subject. Very often values on some biomarkers may be missing. One possible approach to dealing with this situation is to use multiple imputation based on the multinomial distributions and then apply the proposed method to the imputed data. The effectiveness of this approach in controlling the PCS needs further investigation. Moreover, selection bias may occur when the subjects are not randomly selected, and correction for such bias in estimating the PCS is desirable.

## Figures and Tables

**Table 1:** PCS in fixed-size and two-stage designs.

*p*_111_	*p*_110_	*p*_101_	*p*_100_	*p*_011_	*p*_010_	*p*_001_	*p*_000_	*q*_111_	*q*_110_	*q*_101_	*q*_100_	*q*_011_	*q*_010_	*q*_001_	*q*_000_	*ρ*	ρ^*^
0.811	0.035	0.089	0.035	0.016	0.008	0.004	0.002	0.007	0.043	0.007	0.043	0.017	0.033	0.119	0.731	0.950	0.956
0.324	0.190	0.249	0.038	0.167	0.020	0.011	0.003	0.014	0.069	0.086	0.331	0.377	0.040	0.073	0.010	0.733	0.938
0.448	0.092	0.168	0.092	0.107	0.053	0.027	0.013	0.007	0.043	0.007	0.043	0.017	0.033	0.119	0.731	0.861	0.942
0.358	0.163	0.226	0.054	0.152	0.028	0.015	0.005	0.003	0.022	0.009	0.066	0.019	0.056	0.119	0.706	0.836	0.943
0.324	0.190	0.249	0.038	0.167	0.020	0.011	0.003	0.002	0.015	0.009	0.074	0.017	0.067	0.122	0.694	0.828	0.945
0.448	0.092	0.168	0.092	0.107	0.053	0.027	0.013	0.014	0.086	0.014	0.086	0.033	0.067	0.188	0.512	0.821	0.943
0.358	0.163	0.226	0.054	0.152	0.028	0.015	0.005	0.006	0.044	0.019	0.131	0.038	0.113	0.188	0.463	0.795	0.944
0.324	0.190	0.249	0.038	0.167	0.020	0.011	0.003	0.004	0.030	0.019	0.148	0.033	0.133	0.194	0.439	0.786	0.945
0.448	0.092	0.168	0.092	0.107	0.053	0.027	0.013	0.021	0.129	0.021	0.129	0.050	0.100	0.257	0.293	0.788	0.946
0.358	0.163	0.226	0.054	0.152	0.028	0.015	0.005	0.009	0.066	0.028	0.197	0.056	0.169	0.256	0.219	0.761	0.949
0.324	0.190	0.249	0.038	0.167	0.020	0.011	0.003	0.006	0.044	0.028	0.222	0.050	0.200	0.267	0.183	0.752	0.950
0.448	0.092	0.168	0.092	0.107	0.053	0.027	0.013	0.029	0.171	0.029	0.171	0.067	0.133	0.326	0.074	0.760	0.943
0.358	0.163	0.226	0.054	0.152	0.028	0.015	0.005	0.013	0.088	0.038	0.263	0.150	0.150	0.250	0.050	0.740	0.945
0.324	0.190	0.249	0.038	0.167	0.020	0.011	0.003	0.007	0.059	0.037	0.296	0.178	0.156	0.228	0.039	0.735	0.944
0.448	0.092	0.168	0.092	0.107	0.053	0.027	0.013	0.057	0.193	0.057	0.193	0.207	0.043	0.229	0.021	0.754	0.941
0.358	0.163	0.226	0.054	0.152	0.028	0.015	0.005	0.027	0.098	0.080	0.295	0.333	0.042	0.111	0.014	0.738	0.943
0.512	0.082	0.154	0.082	0.091	0.045	0.023	0.011	0.007	0.043	0.007	0.043	0.017	0.033	0.119	0.731	0.875	0.944
0.433	0.144	0.205	0.048	0.129	0.024	0.013	0.004	0.003	0.022	0.009	0.066	0.019	0.056	0.119	0.706	0.852	0.950
0.404	0.168	0.225	0.034	0.142	0.017	0.009	0.002	0.002	0.015	0.009	0.074	0.017	0.067	0.122	0.694	0.843	0.948
0.512	0.082	0.154	0.082	0.091	0.045	0.023	0.011	0.014	0.086	0.014	0.086	0.033	0.067	0.188	0.512	0.833	0.943
0.433	0.144	0.205	0.048	0.129	0.024	0.013	0.004	0.006	0.044	0.019	0.131	0.038	0.113	0.188	0.463	0.807	0.941
0.404	0.168	0.225	0.034	0.142	0.017	0.009	0.002	0.004	0.030	0.019	0.148	0.033	0.133	0.194	0.439	0.798	0.948
0.512	0.082	0.154	0.082	0.091	0.045	0.023	0.011	0.021	0.129	0.021	0.129	0.050	0.100	0.257	0.293	0.798	0.951
0.433	0.144	0.205	0.048	0.129	0.024	0.013	0.004	0.009	0.066	0.028	0.197	0.056	0.169	0.256	0.219	0.771	0.950
0.404	0.168	0.225	0.034	0.142	0.017	0.009	0.002	0.006	0.044	0.028	0.222	0.050	0.200	0.267	0.183	0.762	0.950
0.512	0.082	0.154	0.082	0.091	0.045	0.023	0.011	0.029	0.171	0.029	0.171	0.067	0.133	0.326	0.074	0.768	0.950
0.433	0.144	0.205	0.048	0.129	0.024	0.013	0.004	0.013	0.088	0.038	0.263	0.150	0.150	0.250	0.050	0.749	0.940
0.404	0.168	0.225	0.034	0.142	0.017	0.009	0.002	0.007	0.059	0.037	0.296	0.178	0.156	0.228	0.039	0.743	0.944
0.512	0.082	0.154	0.082	0.091	0.045	0.023	0.011	0.057	0.193	0.057	0.193	0.207	0.043	0.229	0.021	0.761	0.940
0.433	0.144	0.205	0.048	0.129	0.024	0.013	0.004	0.027	0.098	0.080	0.295	0.333	0.042	0.111	0.014	0.746	0.944
0.404	0.168	0.225	0.034	0.142	0.017	0.009	0.002	0.014	0.069	0.086	0.331	0.377	0.040	0.073	0.010	0.741	0.941
0.555	0.075	0.145	0.075	0.080	0.040	0.020	0.010	0.007	0.043	0.007	0.043	0.017	0.033	0.119	0.731	0.885	0.939
0.484	0.131	0.191	0.044	0.114	0.021	0.011	0.004	0.003	0.022	0.009	0.066	0.019	0.056	0.119	0.706	0.862	0.950
0.457	0.153	0.209	0.031	0.125	0.015	0.008	0.002	0.002	0.015	0.009	0.074	0.017	0.067	0.122	0.694	0.854	0.943
0.555	0.075	0.145	0.075	0.080	0.040	0.020	0.010	0.014	0.086	0.014	0.086	0.033	0.067	0.188	0.512	0.841	0.946

## Supplementary Material

More simulation results on PCS in fixed-size and two-stage designs This supplementary table presents more simulation results on the probability of correct selection for the fixed-size and two-stage designs. Numbers in the table further demonstrate the effectiveness of the two-stage procedures in controlling selection error
