# Supplementary material for: Two-stage procedures for selecting the best diagnostic biomarkers
Source: Philos Trans A Math Phys Eng Sci. 2008 Apr 11;366(1874):2293–9. doi: 10.1098/rsta.2008.0032 (PMC3227145; doi:10.1098/rsta.2008.0032)
Supplement: More simulation results on PCS in fixed-size and two-stage designs — This supplementary table presents more simulation results on the probability of correct selection for the fixed-size and two-stage designs. Numbers in the table further demonstrate the effectiveness of the two-stage procedures in controlling selection error [file rsta20080032s01.pdf]

p111	p110	p101	p100	p011	p010	p001	p000	q111	q110	q101	q100	q011	q010	q001	q000	ρ	ρ*
0.811	0.035	0.089	0.035	0.016	0.008	0.004	0.002	0.007	0.043	0.007	0.043	0.017	0.033	0.119	0.731	0.950	0.956
0.324	0.190	0.249	0.038	0.167	0.020	0.011	0.003	0.014	0.069	0.086	0.331	0.377	0.040	0.073	0.010	0.733	0.938
0.448	0.092	0.168	0.092	0.107	0.053	0.027	0.013	0.007	0.043	0.007	0.043	0.017	0.033	0.119	0.731	0.861	0.942
0.358	0.163	0.226	0.054	0.152	0.028	0.015	0.005	0.003	0.022	0.009	0.066	0.019	0.056	0.119	0.706	0.836	0.943
0.324	0.190	0.249	0.038	0.167	0.020	0.011	0.003	0.002	0.015	0.009	0.074	0.017	0.067	0.122	0.694	0.828	0.945
0.448	0.092	0.168	0.092	0.107	0.053	0.027	0.013	0.014	0.086	0.014	0.086	0.033	0.067	0.188	0.512	0.821	0.943
0.358	0.163	0.226	0.054	0.152	0.028	0.015	0.005	0.006	0.044	0.019	0.131	0.038	0.113	0.188	0.463	0.795	0.944
0.324	0.190	0.249	0.038	0.167	0.020	0.011	0.003	0.004	0.030	0.019	0.148	0.033	0.133	0.194	0.439	0.786	0.945
0.448	0.092	0.168	0.092	0.107	0.053	0.027	0.013	0.021	0.129	0.021	0.129	0.050	0.100	0.257	0.293	0.788	0.946
0.358	0.163	0.226	0.054	0.152	0.028	0.015	0.005	0.009	0.066	0.028	0.197	0.056	0.169	0.256	0.219	0.761	0.949
0.324	0.190	0.249	0.038	0.167	0.020	0.011	0.003	0.006	0.044	0.028	0.222	0.050	0.200	0.267	0.183	0.752	0.950
0.448	0.092	0.168	0.092	0.107	0.053	0.027	0.013	0.029	0.171	0.029	0.171	0.067	0.133	0.326	0.074	0.760	0.943
0.358	0.163	0.226	0.054	0.152	0.028	0.015	0.005	0.013	0.088	0.038	0.263	0.150	0.150	0.250	0.050	0.740	0.945
0.324	0.190	0.249	0.038	0.167	0.020	0.011	0.003	0.007	0.059	0.037	0.296	0.178	0.156	0.228	0.039	0.735	0.944
0.448	0.092	0.168	0.092	0.107	0.053	0.027	0.013	0.057	0.193	0.057	0.193	0.207	0.043	0.229	0.021	0.754	0.941
0.358	0.163	0.226	0.054	0.152	0.028	0.015	0.005	0.027	0.098	0.080	0.295	0.333	0.042	0.111	0.014	0.738	0.943
0.512	0.082	0.154	0.082	0.091	0.045	0.023	0.011	0.007	0.043	0.007	0.043	0.017	0.033	0.119	0.731	0.875	0.944
0.433	0.144	0.205	0.048	0.129	0.024	0.013	0.004	0.003	0.022	0.009	0.066	0.019	0.056	0.119	0.706	0.852	0.950
0.404	0.168	0.225	0.034	0.142	0.017	0.009	0.002	0.002	0.015	0.009	0.074	0.017	0.067	0.122	0.694	0.843	0.948
0.512	0.082	0.154	0.082	0.091	0.045	0.023	0.011	0.014	0.086	0.014	0.086	0.033	0.067	0.188	0.512	0.833	0.943
0.433	0.144	0.205	0.048	0.129	0.024	0.013	0.004	0.006	0.044	0.019	0.131	0.038	0.113	0.188	0.463	0.807	0.941
0.404	0.168	0.225	0.034	0.142	0.017	0.009	0.002	0.004	0.030	0.019	0.148	0.033	0.133	0.194	0.439	0.798	0.948
0.512	0.082	0.154	0.082	0.091	0.045	0.023	0.011	0.021	0.129	0.021	0.129	0.050	0.100	0.257	0.293	0.798	0.951
0.433	0.144	0.205	0.048	0.129	0.024	0.013	0.004	0.009	0.066	0.028	0.197	0.056	0.169	0.256	0.219	0.771	0.950
0.404	0.168	0.225	0.034	0.142	0.017	0.009	0.002	0.006	0.044	0.028	0.222	0.050	0.200	0.267	0.183	0.762	0.950
0.512	0.082	0.154	0.082	0.091	0.045	0.023	0.011	0.029	0.171	0.029	0.171	0.067	0.133	0.326	0.074	0.768	0.950
0.433	0.144	0.205	0.048	0.129	0.024	0.013	0.004	0.013	0.088	0.038	0.263	0.150	0.150	0.250	0.050	0.749	0.940
0.404	0.168	0.225	0.034	0.142	0.017	0.009	0.002	0.007	0.059	0.037	0.296	0.178	0.156	0.228	0.039	0.743	0.944
0.512	0.082	0.154	0.082	0.091	0.045	0.023	0.011	0.057	0.193	0.057	0.193	0.207	0.043	0.229	0.021	0.761	0.940
0.433	0.144	0.205	0.048	0.129	0.024	0.013	0.004	0.027	0.098	0.080	0.295	0.333	0.042	0.111	0.014	0.746	0.944
0.404	0.168	0.225	0.034	0.142	0.017	0.009	0.002	0.014	0.069	0.086	0.331	0.377	0.040	0.073	0.010	0.741	0.941
0.555	0.075	0.145	0.075	0.080	0.040	0.020	0.010	0.007	0.043	0.007	0.043	0.017	0.033	0.119	0.731	0.885	0.939
0.484	0.131	0.191	0.044	0.114	0.021	0.011	0.004	0.003	0.022	0.009	0.066	0.019	0.056	0.119	0.706	0.862	0.950
0.457	0.153	0.209	0.031	0.125	0.015	0.008	0.002	0.002	0.015	0.009	0.074	0.017	0.067	0.122	0.694	0.854	0.943
0.555	0.075	0.145	0.075	0.080	0.040	0.020	0.010	0.014	0.086	0.014	0.086	0.033	0.067	0.188	0.512	0.841	0.946

0.484	0.131	0.191	0.044	0.114	0.021	0.011	0.004	0.006	0.044	0.019	0.131	0.038	0.113	0.188	0.463	0.816	0.942
0.457	0.153	0.209	0.031	0.125	0.015	0.008	0.002	0.004	0.030	0.019	0.148	0.033	0.133	0.194	0.439	0.807	0.943
0.555	0.075	0.145	0.075	0.080	0.040	0.020	0.010	0.021	0.129	0.021	0.129	0.050	0.100	0.257	0.293	0.804	0.949
0.484	0.131	0.191	0.044	0.114	0.021	0.011	0.004	0.009	0.066	0.028	0.197	0.056	0.169	0.256	0.219	0.778	0.947
0.457	0.153	0.209	0.031	0.125	0.015	0.008	0.002	0.006	0.044	0.028	0.222	0.050	0.200	0.267	0.183	0.769	0.946
0.555	0.075	0.145	0.075	0.080	0.040	0.020	0.010	0.029	0.171	0.029	0.171	0.067	0.133	0.326	0.074	0.774	0.944
0.484	0.131	0.191	0.044	0.114	0.021	0.011	0.004	0.013	0.088	0.038	0.263	0.150	0.150	0.250	0.050	0.754	0.943
0.457	0.153	0.209	0.031	0.125	0.015	0.008	0.002	0.007	0.059	0.037	0.296	0.178	0.156	0.228	0.039	0.749	0.945
0.555	0.075	0.145	0.075	0.080	0.040	0.020	0.010	0.057	0.193	0.057	0.193	0.207	0.043	0.229	0.021	0.767	0.938
0.484	0.131	0.191	0.044	0.114	0.021	0.011	0.004	0.027	0.098	0.080	0.295	0.333	0.042	0.111	0.014	0.751	0.938
0.457	0.153	0.209	0.031	0.125	0.015	0.008	0.002	0.014	0.069	0.086	0.331	0.377	0.040	0.073	0.010	0.747	0.931
0.598	0.068	0.136	0.068	0.069	0.035	0.017	0.009	0.007	0.043	0.007	0.043	0.017	0.033	0.119	0.731	0.895	0.944
0.534	0.119	0.177	0.040	0.099	0.018	0.010	0.003	0.003	0.022	0.009	0.066	0.019	0.056	0.119	0.706	0.873	0.948
0.511	0.138	0.194	0.028	0.109	0.013	0.007	0.002	0.002	0.015	0.009	0.074	0.017	0.067	0.122	0.694	0.866	0.945
0.598	0.068	0.136	0.068	0.069	0.035	0.017	0.009	0.014	0.086	0.014	0.086	0.033	0.067	0.188	0.512	0.850	0.947
0.534	0.119	0.177	0.040	0.099	0.018	0.010	0.003	0.006	0.044	0.019	0.131	0.038	0.113	0.188	0.463	0.825	0.946
0.511	0.138	0.194	0.028	0.109	0.013	0.007	0.002	0.004	0.030	0.019	0.148	0.033	0.133	0.194	0.439	0.816	0.947
0.598	0.068	0.136	0.068	0.069	0.035	0.017	0.009	0.021	0.129	0.021	0.129	0.050	0.100	0.257	0.293	0.812	0.943
0.534	0.119	0.177	0.040	0.099	0.018	0.010	0.003	0.009	0.066	0.028	0.197	0.056	0.169	0.256	0.219	0.785	0.949
0.511	0.138	0.194	0.028	0.109	0.013	0.007	0.002	0.006	0.044	0.028	0.222	0.050	0.200	0.267	0.183	0.776	0.944
0.598	0.068	0.136	0.068	0.069	0.035	0.017	0.009	0.029	0.171	0.029	0.171	0.067	0.133	0.326	0.074	0.780	0.945
0.534	0.119	0.177	0.040	0.099	0.018	0.010	0.003	0.013	0.088	0.038	0.263	0.150	0.150	0.250	0.050	0.761	0.941
0.511	0.138	0.194	0.028	0.109	0.013	0.007	0.002	0.007	0.059	0.037	0.296	0.178	0.156	0.228	0.039	0.755	0.944
0.598	0.068	0.136	0.068	0.069	0.035	0.017	0.009	0.057	0.193	0.057	0.193	0.207	0.043	0.229	0.021	0.772	0.946
0.534	0.119	0.177	0.040	0.099	0.018	0.010	0.003	0.027	0.098	0.080	0.295	0.333	0.042	0.111	0.014	0.757	0.939
0.511	0.138	0.194	0.028	0.109	0.013	0.007	0.002	0.014	0.069	0.086	0.331	0.377	0.040	0.073	0.010	0.752	0.934
0.662	0.058	0.122	0.058	0.053	0.027	0.013	0.007	0.007	0.043	0.007	0.043	0.017	0.033	0.119	0.731	0.911	0.943
0.610	0.100	0.157	0.033	0.076	0.014	0.008	0.003	0.003	0.022	0.009	0.066	0.019	0.056	0.119	0.706	0.891	0.943
0.591	0.116	0.170	0.023	0.084	0.010	0.005	0.001	0.002	0.015	0.009	0.074	0.017	0.067	0.122	0.694	0.884	0.946
0.662	0.058	0.122	0.058	0.053	0.027	0.013	0.007	0.014	0.086	0.014	0.086	0.033	0.067	0.188	0.512	0.863	0.945
0.610	0.100	0.157	0.033	0.076	0.014	0.008	0.003	0.006	0.044	0.019	0.131	0.038	0.113	0.188	0.463	0.839	0.939
0.591	0.116	0.170	0.023	0.084	0.010	0.005	0.001	0.004	0.030	0.019	0.148	0.033	0.133	0.194	0.439	0.830	0.948
0.662	0.058	0.122	0.058	0.053	0.027	0.013	0.007	0.021	0.129	0.021	0.129	0.050	0.100	0.257	0.293	0.823	0.946
0.610	0.100	0.157	0.033	0.076	0.014	0.008	0.003	0.009	0.066	0.028	0.197	0.056	0.169	0.256	0.219	0.797	0.945
0.591	0.116	0.170	0.023	0.084	0.010	0.005	0.001	0.006	0.044	0.028	0.222	0.050	0.200	0.267	0.183	0.788	0.951
0.662	0.058	0.122	0.058	0.053	0.027	0.013	0.007	0.029	0.171	0.029	0.171	0.067	0.133	0.326	0.074	0.789	0.946

0.610	0.100	0.157	0.033	0.076	0.014	0.008	0.003	0.013	0.088	0.038	0.263	0.150	0.150	0.250	0.050	0.770	0.942
0.591	0.116	0.170	0.023	0.084	0.010	0.005	0.001	0.007	0.059	0.037	0.296	0.178	0.156	0.228	0.039	0.765	0.942
0.662	0.058	0.122	0.058	0.053	0.027	0.013	0.007	0.057	0.193	0.057	0.193	0.207	0.043	0.229	0.021	0.780	0.938
0.610	0.100	0.157	0.033	0.076	0.014	0.008	0.003	0.027	0.098	0.080	0.295	0.333	0.042	0.111	0.014	0.765	0.934
0.591	0.116	0.170	0.023	0.084	0.010	0.005	0.001	0.014	0.069	0.086	0.331	0.377	0.040	0.073	0.010	0.761	0.938
0.726	0.048	0.108	0.048	0.037	0.019	0.009	0.005	0.007	0.043	0.007	0.043	0.017	0.033	0.119	0.731	0.928	0.945
0.686	0.081	0.136	0.027	0.053	0.010	0.005	0.002	0.003	0.022	0.009	0.066	0.019	0.056	0.119	0.706	0.910	0.942
0.671	0.094	0.147	0.019	0.058	0.007	0.004	0.001	0.002	0.015	0.009	0.074	0.017	0.067	0.122	0.694	0.904	0.946
0.726	0.048	0.108	0.048	0.037	0.019	0.009	0.005	0.014	0.086	0.014	0.086	0.033	0.067	0.188	0.512	0.877	0.942
0.686	0.081	0.136	0.027	0.053	0.010	0.005	0.002	0.006	0.044	0.019	0.131	0.038	0.113	0.188	0.463	0.854	0.944
0.671	0.094	0.147	0.019	0.058	0.007	0.004	0.001	0.004	0.030	0.019	0.148	0.033	0.133	0.194	0.439	0.846	0.947
0.726	0.048	0.108	0.048	0.037	0.019	0.009	0.005	0.021	0.129	0.021	0.129	0.050	0.100	0.257	0.293	0.835	0.946
0.686	0.081	0.136	0.027	0.053	0.010	0.005	0.002	0.009	0.066	0.028	0.197	0.056	0.169	0.256	0.219	0.809	0.945
0.671	0.094	0.147	0.019	0.058	0.007	0.004	0.001	0.006	0.044	0.028	0.222	0.050	0.200	0.267	0.183	0.800	0.946
0.726	0.048	0.108	0.048	0.037	0.019	0.009	0.005	0.029	0.171	0.029	0.171	0.067	0.133	0.326	0.074	0.799	0.946
0.686	0.081	0.136	0.027	0.053	0.010	0.005	0.002	0.013	0.088	0.038	0.263	0.150	0.150	0.250	0.050	0.780	0.945
0.671	0.094	0.147	0.019	0.058	0.007	0.004	0.001	0.007	0.059	0.037	0.296	0.178	0.156	0.228	0.039	0.775	0.942
0.726	0.048	0.108	0.048	0.037	0.019	0.009	0.005	0.057	0.193	0.057	0.193	0.207	0.043	0.229	0.021	0.789	0.940
0.686	0.081	0.136	0.027	0.053	0.010	0.005	0.002	0.027	0.098	0.080	0.295	0.333	0.042	0.111	0.014	0.775	0.936
0.671	0.094	0.147	0.019	0.058	0.007	0.004	0.001	0.014	0.069	0.086	0.331	0.377	0.040	0.073	0.010	0.771	0.936
0.768	0.042	0.098	0.042	0.027	0.013	0.007	0.003	0.007	0.043	0.007	0.043	0.017	0.033	0.119	0.731	0.939	0.949
0.736	0.069	0.122	0.023	0.038	0.007	0.004	0.001	0.003	0.022	0.009	0.066	0.019	0.056	0.119	0.706	0.923	0.943
0.725	0.079	0.131	0.016	0.042	0.005	0.003	0.001	0.002	0.015	0.009	0.074	0.017	0.067	0.122	0.694	0.918	0.947
0.768	0.042	0.098	0.042	0.027	0.013	0.007	0.003	0.014	0.086	0.014	0.086	0.033	0.067	0.188	0.512	0.887	0.944
0.736	0.069	0.122	0.023	0.038	0.007	0.004	0.001	0.006	0.044	0.019	0.131	0.038	0.113	0.188	0.463	0.865	0.944
0.725	0.079	0.131	0.016	0.042	0.005	0.003	0.001	0.004	0.030	0.019	0.148	0.033	0.133	0.194	0.439	0.857	0.943
0.768	0.042	0.098	0.042	0.027	0.013	0.007	0.003	0.021	0.129	0.021	0.129	0.050	0.100	0.257	0.293	0.843	0.943
0.736	0.069	0.122	0.023	0.038	0.007	0.004	0.001	0.009	0.066	0.028	0.197	0.056	0.169	0.256	0.219	0.818	0.943
0.725	0.079	0.131	0.016	0.042	0.005	0.003	0.001	0.006	0.044	0.028	0.222	0.050	0.200	0.267	0.183	0.809	0.946
0.768	0.042	0.098	0.042	0.027	0.013	0.007	0.003	0.029	0.171	0.029	0.171	0.067	0.133	0.326	0.074	0.806	0.939
